# Non-Destructive Detection of Soybean Pest Based on Hyperspectral Image and Attention-ResNet Meta-Learning Model

**DOI:** 10.3390/s23020678

**Published:** 2023-01-06

**Authors:** Jiangsheng Gui, Huirong Xu, Jingyi Fei

**Affiliations:** School of Computer Science and Technology, Zhejiang Sci-Tech University, Hangzhou 310018, China

**Keywords:** leguminivora glycinivorella matsumura, A-ResNet, meta-learning, hyperspectral image

## Abstract

Soybean plays an important role in food, medicine, and industry. The quality inspection of soybean is essential for soybean yield and the agricultural economy. However, soybean pest is an important factor that seriously affects soybean yield, among which leguminivora glycinivorella matsumura is the most frequent pest. Aiming at the problem that the traditional detection methods have low accuracy and need a large number of samples to train the model, this paper proposed a detection method for leguminivora glycinivorella matsumura based on an A-ResNet (Attention-ResNet) meta-learning model. In this model, the ResNet network was combined with Attention to obtain the feature vectors that can better express the samples, so as to improve the performance of the model. As well, the classifier was designed as a multi-class support vector machine (SVM) to reduce over-fitting. Furthermore, in order to improve the training stability of the model and the prediction performance on the testing set, the traditional Batch Normalization was replaced by the Layer Normalization, and the Label Smooth method was used to punish the original loss. The experimental results showed that the accuracy of the A-ResNet meta-learning model reached 94.57 ± 0.19%, which can realize rapid and accurate nondestructive detection, and provides theoretical support for the intelligent detection of soybean pests.

## 1. Introduction

Soybean is a widely cultivated plant that provides protein and oil for people [1]. In addition, the demand for soybean is increasing with the growth of the population. Therefore, it is essential to enlarge the yield of soybean. However, due to various reasons such as humidity and temperature, soybeans are prone to breed pests during storage, leading to a decline in quality and price. The main pests that soybean faces during growth and storage are leguminivora glycinivorella matsumura, aphids, leguminous pests [2], etc. Among them, leguminivora glycinivorella matsumura is the most frequent pest. Therefore, the detection of leguminivora glycinivorella matsumura is an urgent task.

Traditional detection methods of crop pests include: biochemical detection [3,4,5], artificial sensory judgment, image processing [6], and spectral data detection [7,8]. Among them, the biochemical detection method is not only destructive to samples, but also time-consuming and high-cost, which is not conducive to large-scale operations. The artificial sensory judgment method has certain subjectivity, and it is difficult to invest a lot of manpower to observe whether there are pests on agricultural products with the naked eyes. The image processing method is not effective for detecting samples with slight damage or inconspicuous images. The spectral data detection method only analyzes the spectral dimension of samples, and lacks the data analysis of spatial dimension, leading to poor detection accuracy. Therefore, traditional detection methods of crop pests are not suitable for pest detection due to their limitations.

In recent years, hyperspectral imaging, as a new nondestructive detection technology, has been proven to be a safety detection technology for crop pests because of its spatial dimension and spectral dimension. The main challenge of detecting crop pests by hyperspectral imaging technology is how to extract and learn effective features. With the rapid development of deep learning, features can be directly extracted from the original hyperspectral images through convolutional neural networks, which avoids the complicated design of traditional methods. At present, hyperspectral image detection methods based on deep learning mainly include spatial feature extraction networks, spectral feature extraction networks and spatial-spectral feature extraction networks. Iost Filho F H et.al [9] explored the deep learning method based on multi-layer perceptron to classify pests in soybean production, and realized high-precision detection. Sulaiman N [10] analyzed the spectral reflectance of weeds and plants, and combined the spectral information of hyperspectral remote sensing images with deep learning modeling to complete the detection of weeds. Yan T et al. [11] extracted spectrum and RGB images of cotton leaves from hyperspectral images for 1D and 2D modeling, and hyperspectral images for 3D modeling, and finally achieved 98% classification accuracy on 1DCNN. In order to improve the performance of samples with a small number of markers, Xi B et al. [12] introduced metric-based learning with few samples into hyperspectral classification. Zuo X et al. [13] proposed a small sample learning algorithm based on an edge table graph neural network to solve the problem of low classification accuracy caused by limited labeled samples in existing hyperspectral classification.

However, although deep learning can extract more comprehensive and deeper features of hyperspectral images, these methods can achieve satisfactory results only if there are enough samples for training the network. When the number of training samples reduces, the detection accuracy of the above methods will decline to different degrees. In practice, there are limited samples of known classes, which limits the learning ability of deep learning models to a certain extent, and makes it difficult to extract typical features from hyperspectral images, thus affecting the detection accuracy of hyperspectral images. Meta-learning algorithm is used to solve the problem of a small number of samples, it makes the algorithm closer to human thinking mode, learns from past tasks, and draws inferences from others. Meta-learning algorithms can be divided into three categories: model-based algorithms, metric-based algorithms, and optimization-based algorithms. Gomes J C et al. [14] explored a prototype model of a few-shot, and used Kullback-Leibler divergence measurement to detect pests in the mature stage and early stage. Finally, the accuracy rate in the mature stage was 86.33%, and that in the early stage was 87.91%. Yang J et al. [15] searched for efficient data from a small amount of data, studied the edge distance-entropy data evaluation method, and got 100% effect when using 60% data, which solved the problem that the algorithm based on deep learning needs a large amount of data to obtain accuracy. From the above research, it can be seen that the meta-learning algorithm can achieve a good detection effect under the condition of small samples, and can realize the detection of diseases and pests in the quality of agricultural products. At present, only a few research works studied the application of the meta-learning algorithm to detect the pests of agricultural products, and there are very few researches that combine hyperspectral imaging technology with the meta-learning idea to detect soybean pests. In this paper, A-ResNet meta-learning model which combined hyperspectral images was established to realize the non-destructive detection of soybean insects.

The main contributions of this paper are as follows:

(1) By combining the ResNet network with Attention, the feature vector which can better express the sample can be obtained to improve the model performance.
(2) The step of feature stitching was abandoned, and the classifier was simplified and designed as a multi-class support vector machine to reduce over-fitting.
(3) In order to optimize the model, and improve the training stability of the model and the prediction performance on the testing set, Layer Normalization was used to replace the traditional Batch Normalization, and the Label Smoothing method was used to punish the original loss.

## 2. Materials and Methods

### 2.1. Sample Preparation

The soybeans and the larvae of leguminivora glycinivorella matsumura used in the experiment were all from the Zhejiang Academy of Agricultural Sciences. First of all, the larvae of leguminivora glycinivorella matsumura were placed in a warm, humid, and dimly lit incubator suitable for their growth, and observed the state of the leguminivora glycinivorella matsumura every day, waiting for it to pupate and grew into an oviposition adult. After the leguminivora glycinivorella, matsumura grew into adults, placing them in an incubator containing soybeans and keeping the temperature at 25 °C, and 20 adults were put into soybean to lay eggs on soybeans. A total of 240 soybean seeds were collected, including 60 normal soybean seeds. After the adults were placed in the incubator containing soybeans for five days, the surface of soybeans contained 60 soybeans with eggs; after fifteen days, there were 60 soybeans containing larvae; after thirty days, the larvae grew into adults and ate 60 soybeans containing wormhole. Hyperspectral images were collected for them respectively.

### 2.2. Hyperspectral Imaging System

**Figure 1:**
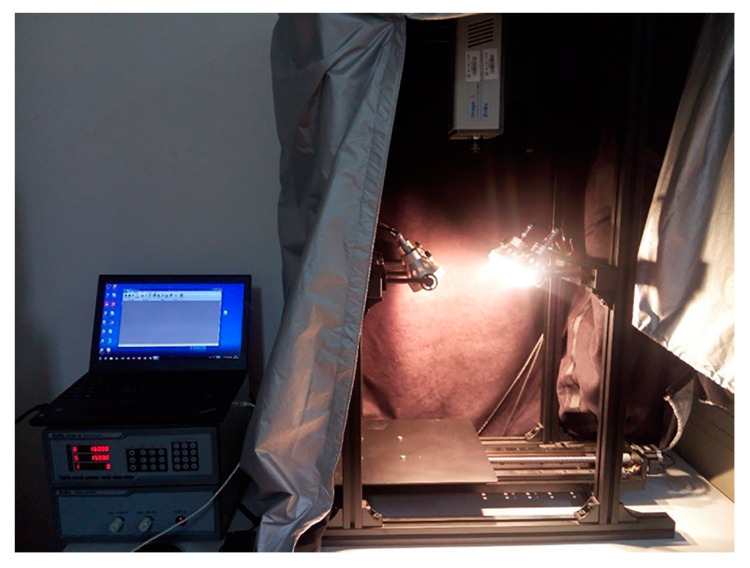
Hyperspectral imaging system.

The hyperspectral imaging system used in the experiment is shown in Figure 1. It mainly included a hyperspectral imager (Imperx IPX-2M30, Sichuan Shuang Li he pu technology co., ltd, Chengdu, China), a CCD camera, an electronically controlled translation stage, four 150 W halogen lamps, and a computer. The collected spectrum ranged from 383.70 nm to 1032.70 nm, including 256 spectral bands, and the spectral resolution was 2.73 nm. The hyperspectral image collecting software was SpectraVIEW Ⅱv1.0.41. In order to avoid the impact of ambient light on the collected images, the entire experimental collection process was completed in a dark box.

### 2.3. Image Collection

**Figure 2:**
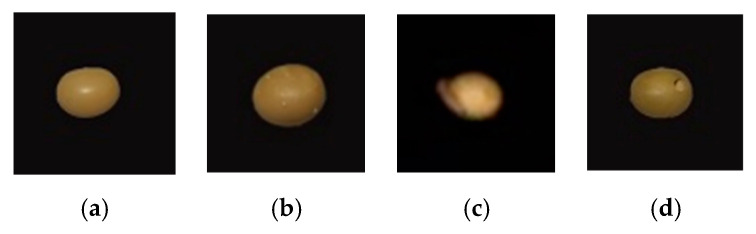
Hyperspectral image of soybean samples ((**a**). Normal soybean; (**b**). Soybean with egg; (**c**). Soybean with larvae; (**d**). Gnawed soybean).

Before collecting hyperspectral images of soybeans, the instrument was preheated for about 30 min to prevent the unstable state when the instrument was just started, and at the same time, eliminate the influence of baseline draft. In SpectraVIEW software, the exposure time of the camera was set to 18 ms, and the displacement speed of the platform was set to 1.50 cm/s, which can prevent the captured image from being distorted or deformed due to the mismatch between the moving speed and the camera acquisition speed. The angle between the 4 halogen lamps and the platform was 50 degrees. After the above parameters were adjusted, a soybean sample was placed on the displacement platform every time to complete the acquisition of a soybean hyperspectral image. The collected soybean samples were shown in Figure 2.

**Figure 3:**
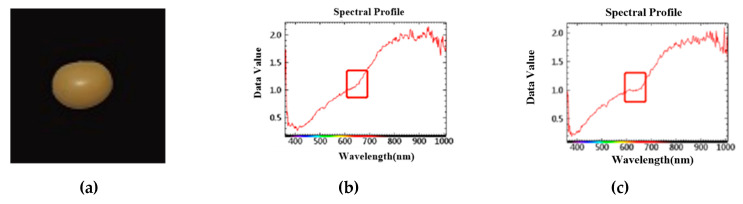
Spectral information of hyperspectral images whose insect information is not obvious in image representation. ((**a**). Two-dimensional image information (**b**). Spectral information of soybean not eaten by insects (**c**). Spectral information of soybean eaten by insects).

When the image representation is not obvious, such as the wormhole on the opposite side of the photographed surface, the hyperspectral image can obtain the information of soybean being wormed from the spectral information. As shown in Figure 3, the spectral information changed at 600~700 nm, and the spectral reflectance of the soybean that was eaten by insects was lower than that of the soybean that was not eaten by insects.

### 2.4. Image Preprocessing

#### 2.4.1. Black-and-White Calibration

In order to avoid the interference of dark current in CCD camera on image acquisition, it is necessary to calibrate soybean hyperspectral image in black and white [16]. First, point the camera at the PTFE (polytetrafluoroethylene) whiteboard, and obtain an image of the whiteboard $R_{\mathrm{white}\;}\left(\lambda \right)$, then screw on the lens cover and scan a blackboard image $R_{\mathrm{dark}\;}\left(\lambda \right)$. The calculation formula of black and white calibration is:

(1) $$I_{xy}\left(\lambda \right)=\frac{R_{xy}\left(\lambda \right)-R_{\mathrm{dark}}\left(\lambda \right)}{R_{\mathrm{white}}\left(\lambda \right)-R_{\mathrm{dark}}\left(\lambda \right)}$$

Among them, $R_{xy}\left(\lambda \right)$ is the original image data; $R_{\mathrm{dark}}\left(\lambda \right)$ is all black image data; $R_{\mathrm{white}}\left(\lambda \right)$ is all white image data; $I_{xy}\left(\lambda \right)$ is the corrected image data.

#### 2.4.2. Region of interest Extraction

**Figure 4:**
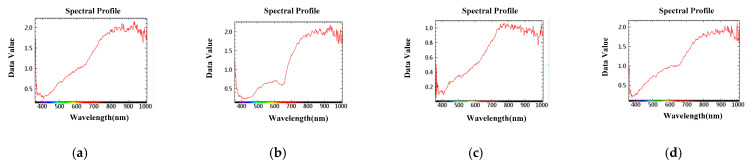
Spectral data of soybean gnawed by insects in different periods. (**a**). Normal soybean; (**b**). Soybean with egg; (**c**). Soybean with larvae; (**d**). Gnawed soybean.

In this research, the center of the sample was taken as the center point of the region of interest, and selected a square with an area of 50 × 50 pixels. As shown in Figure 4, the wave band jitter of soybean samples was large between 800 nm and 1000 nm.

#### 2.4.3. Savitzky–Golay (SG) [17]

In order to reduce the diffuse reflection and zero drift caused by the uneven surface of the sample, which can make the collected image noisy and affect the subsequent model detection results, Savitzky–Golaymethod was used to smooth the spectral dimension of soybean images, where the width of the filter window is $p=2p^{\prime }+1$ and the position of each value to be measured is x=(−p,−p+1,…,0,1,…,p−1,p). An n-1 degree polynomial Equation (2) was used to fit all the values to be measured:

(2) $$y=a_{0}+a_{1}x+a_{2}x^{2}+\cdots +a_{k-1}x^{n-1}$$

The fitting parameter A is determined by least squares fitting, as shown in Equation (3)

(3) $$\left[\begin{array}{c} y_{-p}\\ y_{-p+1}\\ \cdots \\ y_{p} \end{array}\right]=\left[\begin{array}{cccc} 1 & -p & \cdots & {\left(-p\right)}^{n-1}\\ 1 & -p+1 & \cdots & {\left(-p+1\right)}^{n-1}\\ \cdots & \cdots & \cdots & \cdots \\ 1 & p & \cdots & p^{n-1} \end{array}\right]\left[\begin{array}{c} a_{0}\\ a_{1}\\ \cdots \\ a_{n-1} \end{array}\right]+\left[\begin{array}{c} e_{-p}\\ e_{-p+1}\\ \cdots \\ e_{p} \end{array}\right]$$

where $\hat{A}={\left(X^{T}\cdot X\right)}^{-1}\cdot X^{T}\cdot Y$ is the least squares solution of A, and $\hat{Y}=X\cdot \hat{A}=X\cdot {\left(X^{T}\cdot X\right)}^{-1}\cdot X^{T}\cdot Y$ is the predicted value of Y.

**Figure 5:**
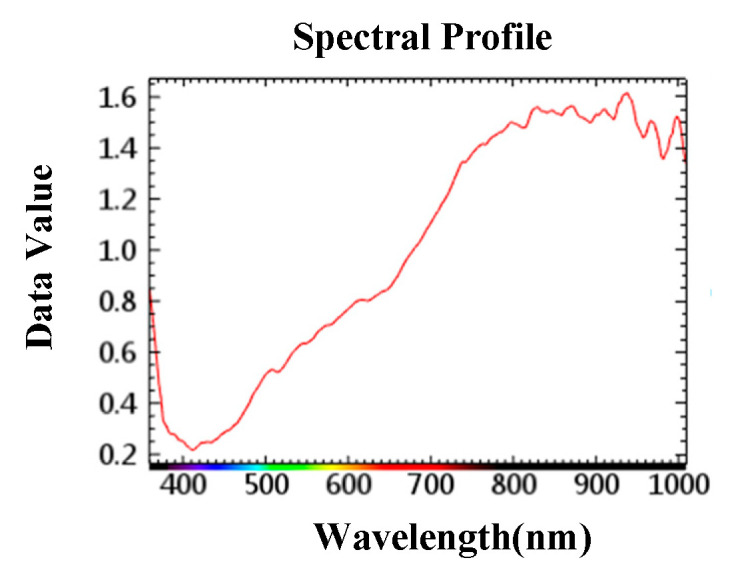
Spectral data of soybean processed by SG filtering.

As can be seen from Figure 5, SG filtering was performed on the extracted spectral information of the region of interest, the band after SG filtering was smoother, and some peaks of the original band were well preserved.

#### 2.4.4. Principal Component Analysis (PCA) [18]

Hyperspectral images are composed of many narrow-band images, and the correlation between the bands is relatively large, which can cause data redundancy and a large number of repeated calculations. Therefore, in this research, Principal Component Analysis was used to reduce the dimensionality of the soybean hyperspectral image. PCA compresses the original spectrum into a linear combination of several orthogonal principal components through data dimensionality reduction, which can eliminate the possible multicollinearity among spectral variables and extract the combination of feature factor that can best represent the original spectrum information without losing important information as much as possible. The formula is:

(4) $$Y=t_{1}p_{1}^{T}+t_{2}p_{2}^{T}+\cdots +t_{k}p_{k}^{T}+E$$

Among them, Y is the spectral matrix of the sample, t is the score matrix, p is the load vector, E is a residual matrix.

In this research, the first 9 hyperspectral bands were selected as the characteristic bands and a 50 × 50 pixel square area was selected as the region of interest with the sample as the center, then the data was converted into 50 × 50 × 9 hyperspectral data.

### 2.5. Methods

#### 2.5.1. Meta-Learning

Meta-learning can quickly learn new tasks according to the acquired knowledge, and make the network have the ability to learn, so as to solve the problem of small samples. The principle of meta-learning is that, it has a prescribed training mode and includes meta-training data and meta-test data, both of which include a support set and a query set. Given an N-way K-shot detection task, the support set contains N classes, and each class contains K-labeled samples. The query set contains N classes and unlabeled samples.

#### 2.5.2. Feature Extraction Network

**Figure 6:**
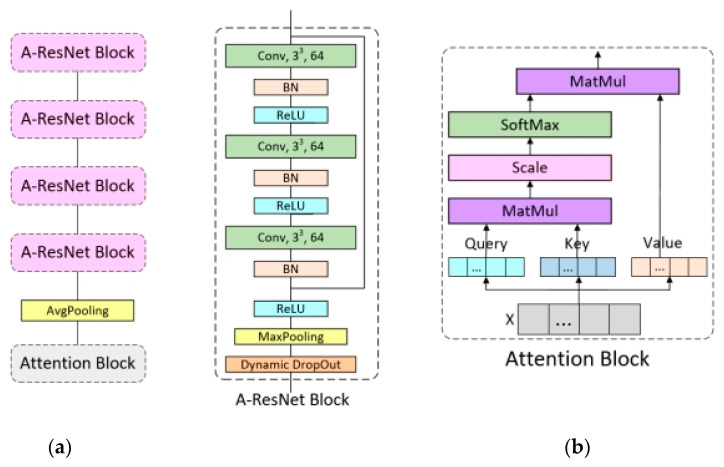
A-ResNet network structure. (**a**) is the overall structure of the A-ResNet network; (**b**) is the Attention Block.

The feature extraction network was A-ResNet, which combined residual network with the attention mechanism. For the residual block, it is composed of three layers of convolution blocks. The first two convolution blocks contain 3D convolution layer, normalization layer and ReLU activation function, while the last convolution block contains a 3D convolution layer and normalization layer. The input data of the first convolution layer is added with the normalized output data of the third convolution layer, and the obtained data is input to the ReLU activation function, the maximum pool layer and dynamic Dropout, which are used to reduce the amount of data. After the data is processed by four residual blocks, it is input into the Attention module. The structure of A-ResNet is shown in Figure 6a.

Attention is a module to find the feature area in the sample that needs the most attention by interacting the features of various dimensions between samples. The input data of Attention contains three vectors, which are query (Query), key (Key) and value (Value) vectors, which are a query to a series of key-value pairs mapping. The feature vector that needs attention finally output is the product of the feature of each dimension of the sample and the attention weight of the feature. The steps of attention are as follows:

(1) Attention first initializes three different weight matrices for the input vector, and multiplies the input data by the above three weight matrices to obtain three input vectors of the same latitude of Query, Key and Value.
(2) In order to enable the model to learn the attention scores of different dimensions of the sample features, multiply the Query and Key values to calculate the attention scores of each dimension feature. The formula is:
  (5) score= Query × Key 

Normalize the calculated attention score and convert the score into probability form via SoftMax, the formula is:

(6) $$\mathrm{value}\;=\mathrm{softmax}\left(\frac{\mathrm{Query}\times {\mathrm{Key}}^{T}}{\sqrt{d_{\mathrm{key}}}}\right)$$

(3) Finally, multiply the value with Value to obtain the final weighting matrix A. The structure of Attention is shown in Figure 6b.

#### 2.5.3. Multi-Class SVM Classifier

The classifier in this study is a multi-class support vector machine. The objective function of support vector is the convex function. Under the condition of a small sample, convex function can perform meta-learning classification tasks well. The implicit differentiability of the convex function can obtain the global optimal solution by using the off-line convex optimization method, and the parameters to be optimized under the condition of the small sample are far smaller than the characteristic dimension, so the model performance can be improved. For K-type linear SVM objective function parameter values θ, the formula is:

(7) $$\begin{array}{l} \theta =\{\omega_{k}{\}}_{k=1}^{K}=S(D^{train};\phi )=\arg\min_{\omega_{k}}\min_{\left\{\xi_{i}\right\}}\frac{1}{2}\sum_{k}∥\omega_{k}{∥}_{2}^{2}+C\sum_{n}\xi_{n}\\ \left(\omega_{y_{n}}\cdot f_{\phi }(x_{n})-\omega_{k}\cdot f_{\phi }(x_{n})\geq 1-\delta_{y_{n},k}-\xi_{n},\forall n,k\right) \end{array}$$

Among them, $D^{train}=\{(x_{n},y_{n})\}$, *C* is the regularization parameter, ϕ is the parameter of the A-ResNet model, and δ is the Kronecker function.

The objective function formula of the Multi-class SVM model is:

(8) $$L^{meta}(D^{test};\theta ,\phi ,\gamma )=\sum_{(x,y)\in D^{test}}\left[-\gamma \omega_{y}\cdot f_{\phi }(x)+\log\sum_{k}\exp(\gamma \omega_{k}\cdot f\phi (x))\right]$$

Among them, θ is the parameter value obtained by Formula (7), and γ is a learnable scale parameter.

#### 2.5.4. A-ResNet Meta-Learning Model

A-ResNet meta-learning model is a meta-learning method based on model optimization, which can achieve the minimum generalization error for different learning tasks under the condition of small samples. A-ResNet meta-learning model is composed of feature extraction network A-ResNet and base learners. A-ResNet maps the input domain to the feature space, and the base learners map from the feature space to the corresponding tasks. By learning A-ResNet, the base learners can have good generalization ability in different tasks.

**Figure 7:**
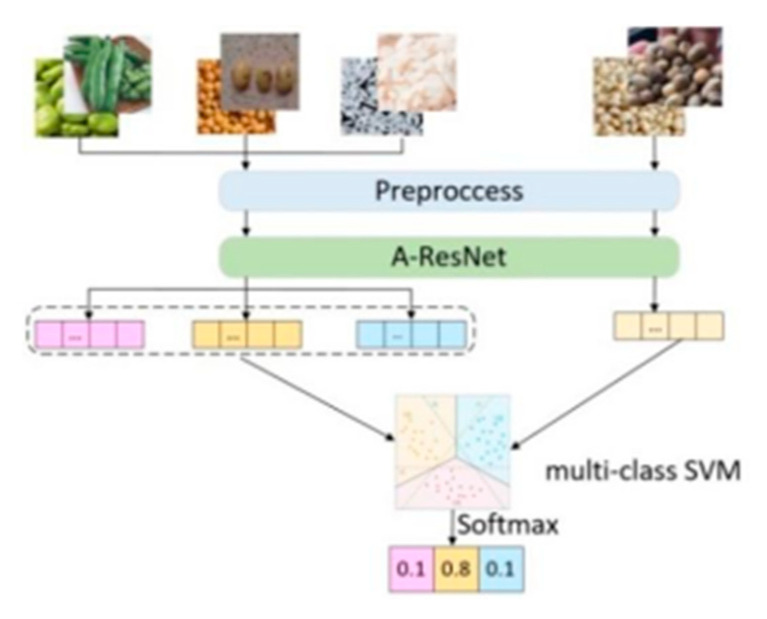
The structure diagram of A-ResNet meta-learning model.

This research divided the dataset into multiple tasks p(T), and each task contained a support set $S={\left\{(D_{i}^{train},D_{i}^{test})\right\}}_{i=1}^{k}$ and a query set $Q={\left\{(D_{j}^{train},D_{j}^{test})\right\}}_{j=1}^{n}$. The samples of the support set and the query set were calculated by inputting the three-dimensional feature extraction network A-ResNet network to obtain the image characteristics $f(x_{i}),f(x_{j})$, and then the average value of the characteristics of the various support set samples was calculated to obtain the prototype representations of the various samples, and then the prototype representations of the various types were compared with the feature vector of the query set sample and input into the support vector machine for detection, finally, the probability of the query set sample corresponding to different classes was output through the Softmax function, and obtain the final detection result. The structure of A-ResNet meta-learning model is shown in Figure 7.

### 2.6. The Optimization of A-ResNet Meta-Learning Model

In order to simplify the model, reduce over-fitting, and improve the stability of the model training and the prediction performance of the testing set, the model needs to be optimized. The optimization methods used in this paper were Layer Normalization, Dropout, and Label Smooth.

#### 2.6.1. Layer Normalization [19]

Independent distribution can speed up the training of the neural network model and improve the prediction ability of the model. With the continuous superposition of neural network layers, the update of each layer’s parameters will lead to the change of the input parameters of the upper layer, which will make the input data of the upper layer no longer present independent and identical distribution, thus leading to the reduction of the learning rate and the early stopping of the model. In order to solve this problem, Batch Normalization is usually used. However, Batch Normalization is sensitive to the size of the batch, and the object of this study is small sample, the effect of Batch Normalization will be very poor when batch is small. Therefore, in order to solve the disadvantages of Batch Normalization, Layer Normalization is introduced. Layer Normalization is to normalize all neuron nodes of a single sample at each layer, and the formula is as follows:

(9) $$\mu^{l}=\frac{1}{H}\overset{H}{\underset{i=1}{\sum }}a_{i}^{l}\sigma^{l}=\sqrt{\frac{1}{H}\overset{H}{\underset{i=1}{\sum }}{\left(a_{i}^{l}-\mu^{l}\right)}^{2}}$$

**Figure 8:**
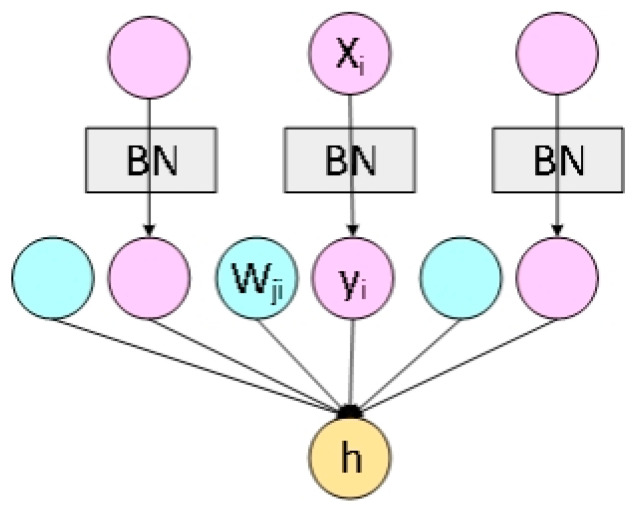
The structure of Batch Normalization.

**Figure 9:**
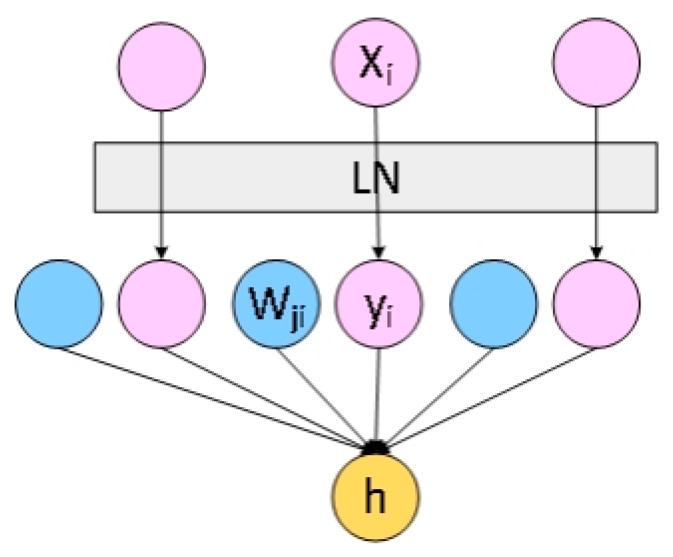
The structure of Layer Normalization.

Inputs of neurons in the same layer in Layer Normalization have the same mean and variance, and different input samples have different mean and variance. Therefore, Layer Normalization does not depend on the size of batch, which is more suitable for the training of small sample learning. The structure of Batch Normalization and Layer Normalization are shown in Figure 8 and Figure 9 respectively.

#### 2.6.2. Inactivation Strategy of Dropout Neurons [20]

**Figure 10:**
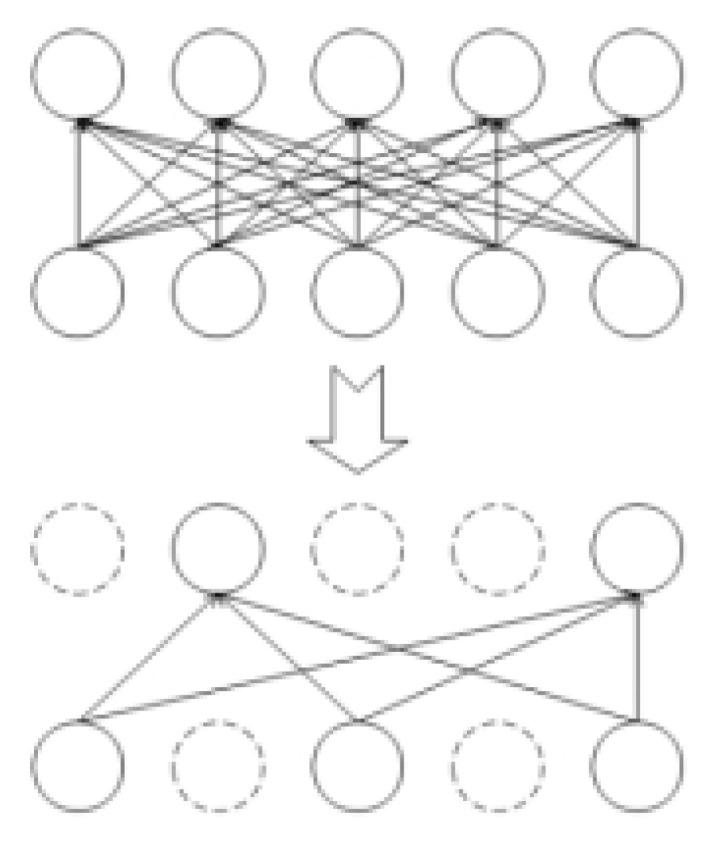
The diagram of Dropout.

The model has many parameters, and this study is a small sample study, so the trained neural network model is easy to over-fit with few training samples. In order to solve the problem of model over-fitting in the background of small samples and simplify the model, the strategy of neuron inactivation by Dropout was adopted. The Dropout makes neurons deactivate in a certain layer with a certain probability, that is, the probability of all neurons in this layer being removed is p. The inactivation of neurons only occurs in the training stage, and all neurons were active in the testing stage, thus achieving the purpose of avoiding over-fitting. In order to make up for the network information removed by this layer in the training stage, the 1-p probability is used to increase the weight, and finally improves the generalization ability of the model. The diagram of Dropout is shown in Figure 10.

#### 2.6.3. Label Smooth [21]

A-ResNet loss function is a cross-entropy loss function. Convolution network will make itself learn in the direction of large error values of correct labels and wrong labels. When there is a small amount of data, it is easy to cause the over-fitting of the network, which makes the adaptability of the network decline. In this paper, the Label Smooth method was used to reduce the weight of the labels of real samples when calculating the loss function, so that the network can suppress the over-fitting when calculating the loss value.

The formal of Label Smooth learning label encoding form is as follows:

(10) $$y_{i}=\{\begin{array}{l} 1-\varepsilon ,i=true\\ \frac{\varepsilon }{K-1},otherwise \end{array}$$

Among them, ε = 0.1, K = 4, which corresponds to K categories in this research and ε is a hyperparameter. The probability of 1−ε in the new label come from the original distribution, and the probability of ε comes from the uniform distribution. Label Smooth changed the form of the original classification task. The original classification target was one-hot target coding. After Label Smooth, the coding bit value of 1 was converted to 1−ε, while the coding bit value of 0 was converted to ε/(K − 1).

## 3. Results and Analysis

### 3.1. Dataset

In this experiment, we used CAVE [22], iCVL [23] and NUS [24] datasets as training datasets. The CAVE dataset is a multispectral dataset collected by Columbia University with 32 scenes. The iCVL dataset is a hyperspectral dataset collected by the European Computer Vision Conference. The dataset covers indoors, parks, plants, rural areas, and cities. The NUS dataset is a hyperspectral dataset containing two classes: general scenes and fruits. The three types datasets were used as meta-training set, and the CutMix [25] method was applied to increase the data of the meta-training set. The four types of soybean sample data collected in this paper were used as the target test dataset.

### 3.2. Experimental Results and Analysis

**Figure 11:**
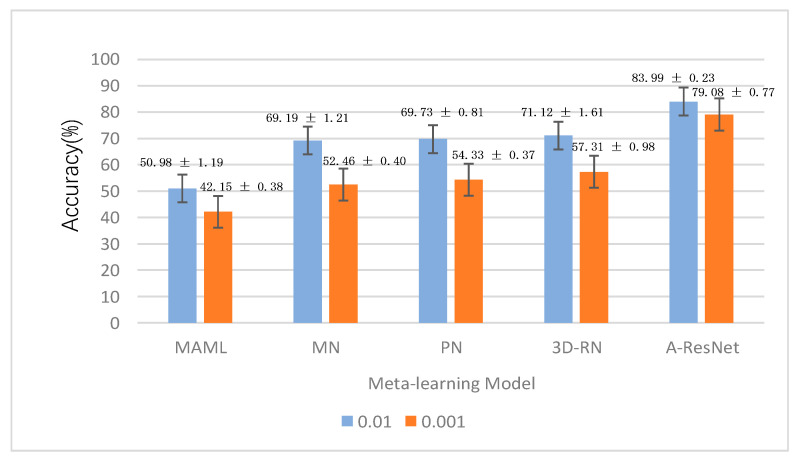
The accuracy of the A-ResNet model and other models under the 1-shot case.

**Figure 12:**
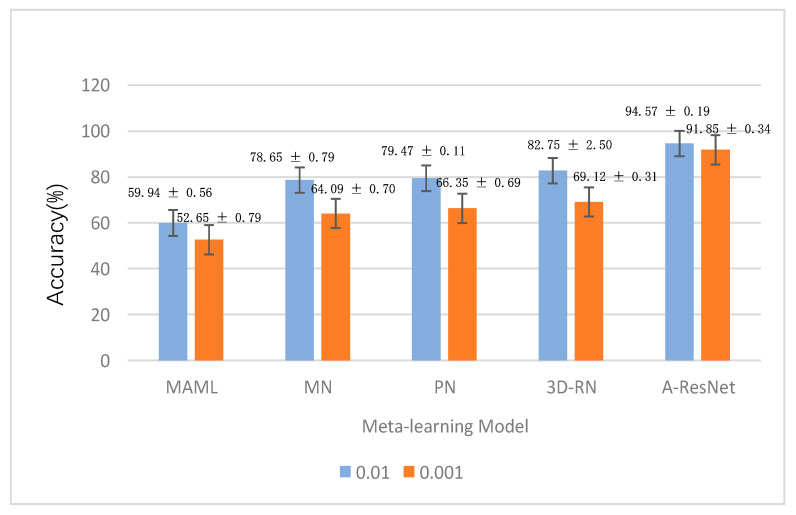
The accuracy of the A-ResNet model and other models under the 5-shot case.

In this research, the performance of A-ResNet meta-learning model in detecting leguminivora glycinivorella matsumura was evaluated. In this experiment, the learning parameter C in the multi-class support vector machine was set to 0.1, the number N of classes in the support set and query set is set to 4 (4-way), and the number K of each class in the support set is divided into two experiments: 1 sample (1-shot) and five samples (5-shot). In order to explore the influence of learning rate on the experimental results, experiments with learning rates of 0.01 and 0.001 were added in the experiment. We compared our model with MAML [26], MN [27], PN [28], and 3D-RN [29] meta-learning models, and analyzed the experimental results. The experimental results are shown in Figure 11 and Figure 12.

As can be seen from Figure 11 and Figure 12, the following conclusions can be drawn from the experimental results:

(1) Under the same shot, the accuracy of the same model with the learning rate of 0.01 was higher than 0.001, especially in MN, PN, and 3D-RN meta-learning models, with the difference of accuracy higher than 10%. This phenomenon showed that this kind of model was greatly influenced by the learning rate hyperparameter. When the learning rate was low, the loss function of the model changed slowly, so it stayed at the local optimal saddle point in advance.
(2) It can be seen that the detection result of 5-shot was always better than that of 1-shot no matter what model, and the accuracy of A-ResNet model achieved the highest accuracy of 94.57% ± 0.19% in the case of 5-shot, which was better than the 3D-RN model, MAML, MN and PN models. It showed that when the number of test samples was large, the model can better learn the feature vectors representing the characteristics of the samples, thus improving the detection performance.
(3) The effect of large learning rate was always better than that of small learning rate, which indicated that when the sample size was small, the small learning rate will lead to the slow convergence of the model, resulting in the decline of the model performance.
(4) The performance of multi-class SVM classifier was better than that of using convolution as classifier, which indicated that the nonlinear classifier may cause over-fitting in the case of small sample, while the multi-class SVM linear classifier combined with Label Smooth method can effectively avoid over-fitting and improve the performance of the model, and the stability of the model was better than that of other models.

## 4. Conclusions

At present, hyperspectral imaging technology has been widely used in the detection of agricultural pests and diseases, but it still faces great challenges for the detection of small samples. In this paper, hyperspectral imaging technology and a meta-learning algorithm were combined to establish an A-ResNet model, which was used to realize the nondestructive detection of soybean eaten by leguminivora glycinivorella matsumura. The experimental results showed that, compared with the MAML, MN, PN, and 3D-RN meta-learning models, the detection effect of the A-ResNet model was more accurate, and the final accuracy was 94.57% ± 0.19% in the 5-shot case. The experiment in this paper realized high-precision detection under small samples, and provided a new idea for the intelligent detection of soybean pests.

## Data Availability

The research doesn’t involve humans.
